# Mutations at the hydrophobic core affect Hal3 trimer stability, reducing its Ppz1 inhibitory capacity but not its PPCDC moonlighting function

**DOI:** 10.1038/s41598-018-32979-x

**Published:** 2018-10-02

**Authors:** Carlos Santolaria, Diego Velázquez, Erick Strauss, Joaquín Ariño

**Affiliations:** 1 Departament de Bioquímica i Biologia Molecular and Institut de Biotecnologia i Biomedicina, Universitat Autònoma de Barcelona, Bellaterra, 08193 Barcelona Spain; 2 Department of Biochemistry, Stellenbosch University, Matieland, 7602 South Africa

## Abstract

*S*. *cerevisiae* Hal3 (ScHal3) is a moonlighting protein that, is in its monomeric state, regulates the Ser/Thr protein phosphatase Ppz1, but also joins ScCab3 (and in some instances the Hal3 paralog Vhs3) to form an unusual heterotrimeric phosphopantothenoylcysteine decarboxylase (PPCDC) enzyme. PPCDC is required for CoA biosynthesis and in most eukaryotes is a homotrimeric complex with three identical catalytic sites at the trimer interfaces. However, in *S*. *cerevisiae* the heterotrimeric arrangement results in a single functional catalytic center. Importantly, the specific structural determinants that direct Hal3’s oligomeric state and those required for Ppz1 inhibition remain largely unknown. We mutagenized residues in the predicted hydrophobic core of ScHal3 (L403–L405) and the plant *Arabidopsis thaliana* Hal3 (AtHal3, G115–L117) oligomers and characterized their properties as PPCDC components and, for ScHal3, also as Ppz1 inhibitor. We found that in AtHal3 these changes do not affect trimerization or PPCDC function. Similarly, mutation of ScHal3 L403 has no effect. In contrast, ScHal3 L405E fails to form homotrimers, but retains the capacity to bind Cab3—explaining its ability to rescue a *hal3 vhs3* synthetically lethal mutation. Remarkably, the L405E mutation decreases Hal3’s ability to interact with and to inhibit Ppz1, confirming the importance of the oligomer/monomer equilibrium in Hal3’s Ppz1 regulating function.

## Introduction

The *Saccharomyces cerevisiae SIS2*/*HAL3* gene was simultaneously identified as a suppressor of the *sit4* deletion^1^ and a regulator of salt tolerance^2^. These apparently unrelated functions were unified when it was demonstrated that Hal3 acts as a negative regulatory subunit of the Ppz1 Ser/Thr protein phosphatase^3–5^ through its binding to the catalytic C-terminal domain of the phosphatase^3^. Subsequent work identified the *VHS3* gene, a *HAL3* paralog arising from whole genome duplication, as a second inhibitory subunit of Ppz1. Vhs3 also binds and inhibits Ppz1 *in vitro*, although its physiological role is much less relevant^6^. Ppz1 has a primary role in regulating monovalent cation homeostasis in two different ways: by blocking the influx of potassium through the high-affinity Trk potassium transporters, and by inhibiting the efflux of sodium through downregulation of the expression of the *ENA1* ATPase^5,7,8^. Thus, deletion of *HAL3* results in sensitivity to Na^+^ and Li^+^ cations, whereas overexpression of the gene confers hypertolerance. Similarly, overexpression of Hal3 also has a detrimental effect in the presence of the cell wall stressor caffeine (known to activate the cell wall integrity pathway^9^) in wild type cells and leads to cell lysis in a *slt2* mutant. This is explained by the excessive K^+^ influx and increase in cellular turgor due to the inhibition of Ppz1^10^.

Ppz1-like enzymes are found only in fungal species^11^. However, orthologs of *HAL3* have been identified both in prokaryotic and eukaryotic organisms. This ubiquitous distribution was explained by the fact that ScHal3 (and ScVhs3) are moonlighting proteins, with additional functions distinct of their Ppz1-regulation activity. Specifically, two molecules of ScHal3 (and/or ScVhs3) were shown to associate with a constant ScCab3 subunit (which is also a ScHal3 and ScVhs3 paralog) to form an active, heterotrimeric phosphopantothenoylcysteine decarboxylase (PPCDC) enzyme^12^. PPCDC catalyzes a key decarboxylation step in CoA biosynthesis, which explains the essential nature of *CAB3* and the synthetically lethal phenotype of the *hal3 vhs3* deletions^6,12^. It must be noted that the PPCDC active site is located at the interface of the subunits of the oligomeric enzyme, and involves a FMN molecule as cofactor. On the basis of the 3D structures of *A*. *thaliana* AtHal3a^13,14^ and *Homo sapiens* HsCoaC^15^ orthologs it was proposed that in the active *S*. *cerevisiae* PPCDC holoenzyme ScCab3 provides an essential Cys478 and a conserved Asn442 residue. These residues are necessary for catalysis and binding of the carboxylate of the substrate PPC, respectively. The other key component of the catalytic site, an essential His residue, must be supplied by ScHal3 (His378) or ScVhs3 (His466), since the equivalent His residue in ScCab3 (His391) is not functional^12^. Therefore, a single ScHal3, ScVhs3 or ScCab3 polypeptide chain would be ineffective to provide the decarboxylase activity. In spite of this, it has been observed that ScHal3 itself retains the ability to spontaneously form trimers^16–18^.

The *S*. *cerevisiae* PPCDC subunit composition can be regarded as exceptional, since in most eukaryotic organisms, such as *A*. *thaliana*, PPCDC is an homotrimer made up of much smaller subunits, specifically lacking the N-terminal extension and the acidic C-terminal tail that is found in certain fungal orthologs, such as *C*. *albicans*^19^. In ScHal3, the central domain, denoted as ScHal3 PD (for PPCDC Domain), is required for both PPCDC activity and Ppz1 binding and regulation, although the acidic C-terminal tail also plays an important role for the latter function^16,20^.

**Figure 1:**
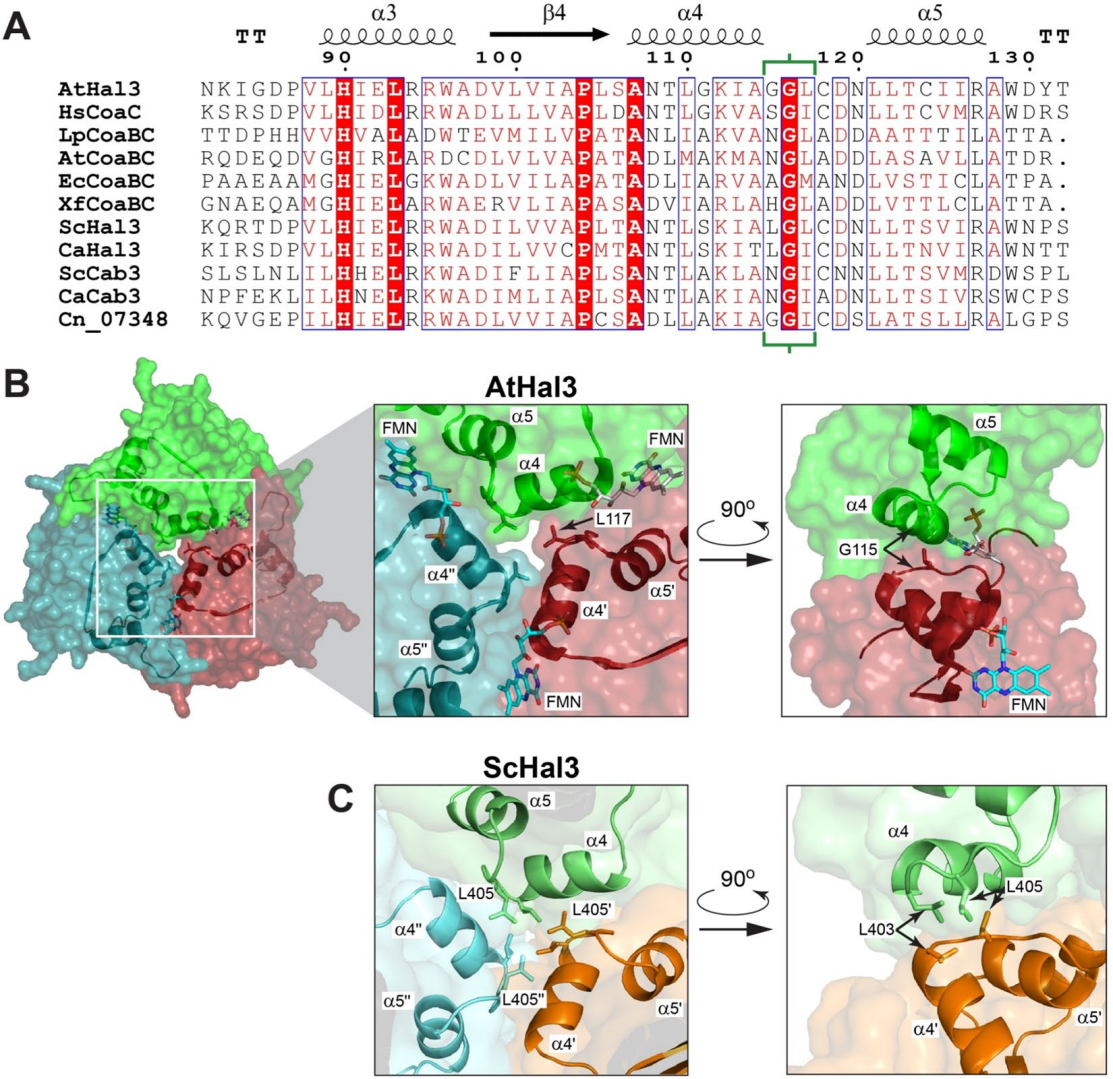
Comparative analysis of the structural context of the AtHal3 and ScHal3 residues modified in this work. (**A**) Sequence alignment of diverse PPCDC and Hal3-like proteins with respect to residues 82–132 (residue numbers indicated above the sequence) of AtHal3a (UniProt accession # Q9SWE5), with the corresponding secondary structure elements of the AtHal3 structure (PDB id 1MVN) indicated. The three residues that formed the focus of this study are bracketed in green. Sequences are taken from human CoaC (HsCoaC; Q96CD2), *Lactobacillus plantarum* CoaBC (LpCoaBC, D7V924), *Agrobacterium tumefaciens* CoaBC (AtCoaBC, F7U4A8), *Escherichia coli* CoaBC (EcCoaBC, P0ABQ0), Xylella fastidiosa CoaBC (XfCoaBC, Q87F20), *S*. *cerevisiae* Hal3 (ScHal3, P36024), *C*. *albicans* Hal3 (CaHal3, A0A1D8PKD2), *S*. *cerevisiae* Cab3 (ScCab3, P36076), *C*. *albicans* Cab3 (CaCab3, Q5A868), *C*. *neoformans* CNAG_07348 (Cn_07348, J9VGI2). (**B**) Structure of the AtHal3 trimer as defined by X-ray crystallography (PDB accession numbers 1MVN), with each of the monomers shown in a different color. The section corresponding to the sequence in panel A is depicted as cartoon to highlight its position in the overall structure, and its involvement in maintaining the central part of the trimer interface. Two zoomed views of the central interface are shown, indicating the positions of the α4 and α5 helices of each monomer, as well as the position of L117 and G115 (shown in stick format). The structure of the FMN cofactor is shown in stick format, colored by atom. Note that in the zoomed view on the right, one of the monomers is not shown for the sake of clarity. (**C**) Two zoomed views of the equivalent region of a model of the ScHal3 trimer built based on the AtHal3 structure. The monomers are shown in different colors, and the identity of the helices is indicated. The L403 and L405 residues of the various chains are shown in stick format and indicated.

While the mechanism of the PPCDC catalytic reaction has been explored in detail^21–25^, little is known about the structural determinants required for formation of Hal3 trimers in *A*. *thaliana* or *S*. *cerevisiae*, as well as the specific elements in ScHal3 that are necessary for its inhibitory role on Ppz1. Previous studies on the interaction between Ppz1 and Hal3 suggested a 1:1 stoichiometry, and provided evidence that Hal3 could easily dissociate from the PPCDC heterotrimeric enzyme to bind Ppz1^18^. Inspection of the 3D structure of Athal3 (PDB accession numbers 1E20 and 1MVN) and of models created for the PD domain of ScHal3, together with alignment of PPCDC sequences from bacteria to human pointed to residues G115 and L117 in AtHal3, and the corresponding L403 and L405 in ScHal3, as possible components of a hydrophobic core that could be important for generating and/or maintaining the trimer (Fig. 1A,B). Therefore, we decided to mutagenize these residues in both the plant and yeast proteins and characterize their properties as PPCDC components and, in the case of ScHal3, their relevance to Ppz1 inhibition. Our results indicate that in AtHal3 residues G115 and L117 are not relevant for trimerization, and their mutation do not affect PPCDC function. In contrast, while mutation of ScHal3 L403 has no effect, change of L405 to Glu alters the ability to form standard trimers and decreases the capacity of this variant to inhibit Ppz1. However, such mutated versions retain the ability to interact *in vit*ro with Cab3 and its expression in yeast rescues the *hal3 vhs3* synthetically lethal phenotype, indicating a fruitful interaction with Cab3 to form a functional PPCDC enzyme.

## Results

### Structural evaluation of AtHal3 and ScHal3 trimeric interfaces

The alignment of multiple PPCDC and diverse Hal3 and Hal3-like yeast proteins ant their mapping onto the AtHal3 3D structure indicates that a large portion of the interface between the subunits is formed by interaction of helices α4 and α5 (Fig. 1A,B). In particular, three of the residues that form part of the link between α4 and α5 of each monomer (115–117 in AtHal3 and 403–405 in ScHal3; region denoted by green brackets in Fig. 1A) are positioned close to one another in the central space created by the association of the three monomers. Of note is that position 117 in AtHal3 (405 in ScHal3) is strongly conserved, usually containing a bulky hydrophobic residue (mostly L or I). This suggested that it might be important for the formation of a hydrophobic core relevant for generation and/or maintenance of the trimer. Position 116 is invariably occupied by Gly, most likely due to constraints imposed by the loop, whereas the residue in position 115 (403 in ScHal3) was more variable, containing Gly, Ala, Leu, Asn or—in a single example—His. In the case of ScHal3, the loop region consists of a combination of Leu Gly Leu, which in a model based on the structure of AtHal3 should result in the formation of a tightly packed hydrophobic core (Fig. 1C). Based on this analysis, we considered that these residues could play a central role in the trimerization/monomerization process and, therefore, in the formation of the functional PPCDC enzyme and the active Ppz1 phosphatase inhibitor. We therefore set out to explore this by means of comparative mutational analysis.

### Analysis of AtHal3 mutants

**Figure 2:**
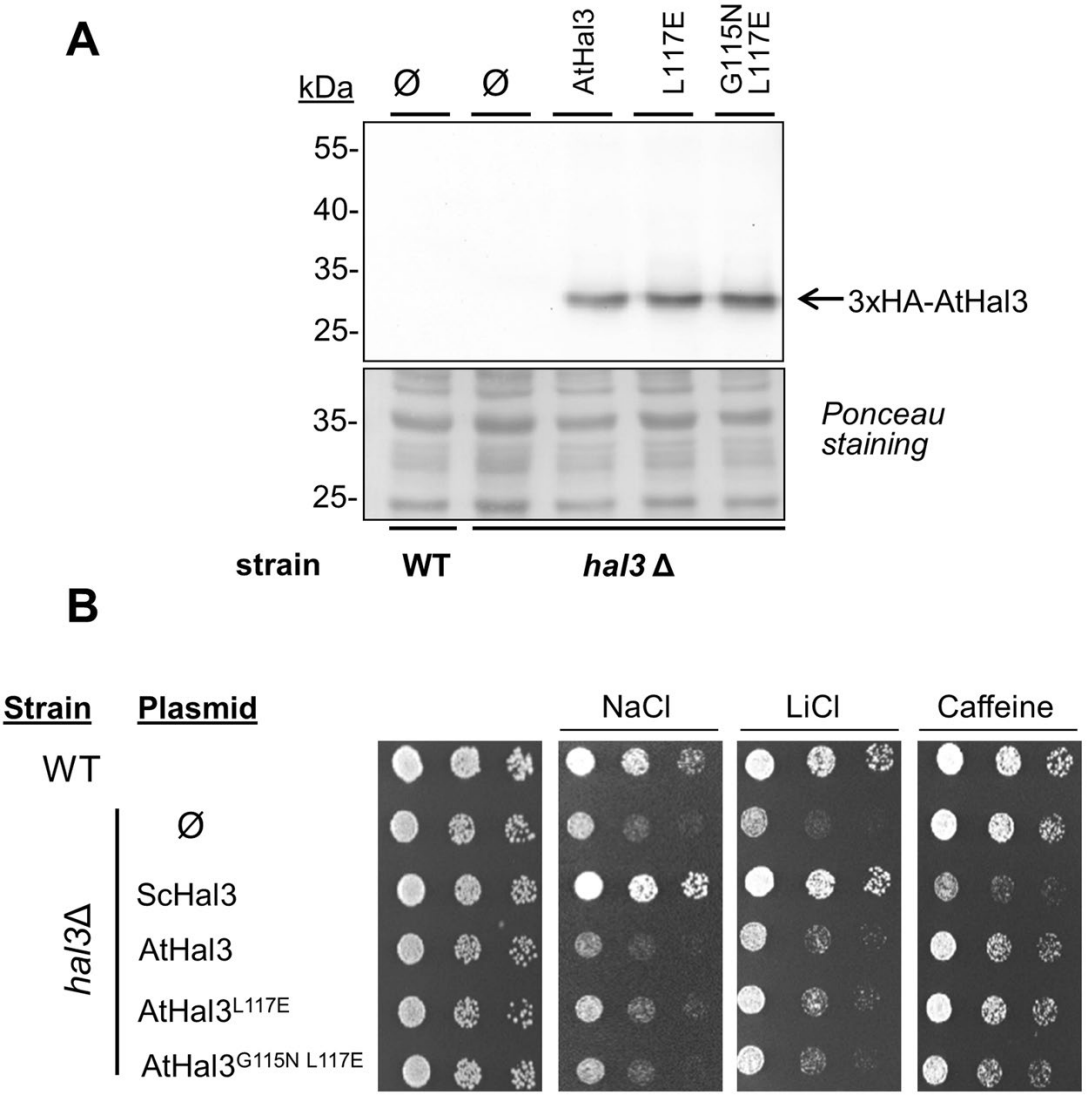
Immunodetection and phenotypic characterization of AtHal3 variants expressed in *S*. *cerevisiae*. (**A**) Protein extracts from cultures of wild type strain BY4741 (WT) and its *hal3Δ* derivative, transformed with plasmid pWS93 (Ø) and the same plasmid expressing AtHal3a or the indicated variants, were prepared and subjected to SDS-PAGE. Proteins were transferred to membranes and the different 3xHA-tagged proteins detected with anti-HA antibodies. Ponceau staining of the membranes is shown for comparison of loading and transfer efficiency. (**B**) Wild type (WT) or *hal3*Δ strains containing the indicated plasmids were spotted at OD_660_ = 0.05 and at 1/5 dilutions on synthetic medium plates lacking uracil and containing NaCl (1 M), LiCl (150 mM) or caffeine (8 mM). Plates were grown for 48 h.

Following the rationale described above we mutagenized AtHal3’s Leu117 to Glu, a residue that is similar in size but polar and negatively charged under physiological conditions, with the objective of disrupting any hydrophobic interactions in which this residue could be involved. In addition, we created a second version in which the Gly at position 115 was changed to the bulkier residue Asn, which should impact on the folding of the loop region. Both variants were expressed in *S*. *cerevisiae* at a level very much like that of the wild type protein (Fig. 2A). Similar to what was previously reported^26^, overexpression of native AtHal3 was found to provide a slight increase in tolerance to toxic Li^+^ cations (Fig. 2B), and this characteristic was shared by both mutated versions. Neither native AtHal3 nor its variants mimicked the harmful effect of ScHal3 overexpression in the presence of small amounts of caffeine.

**Figure 3:**
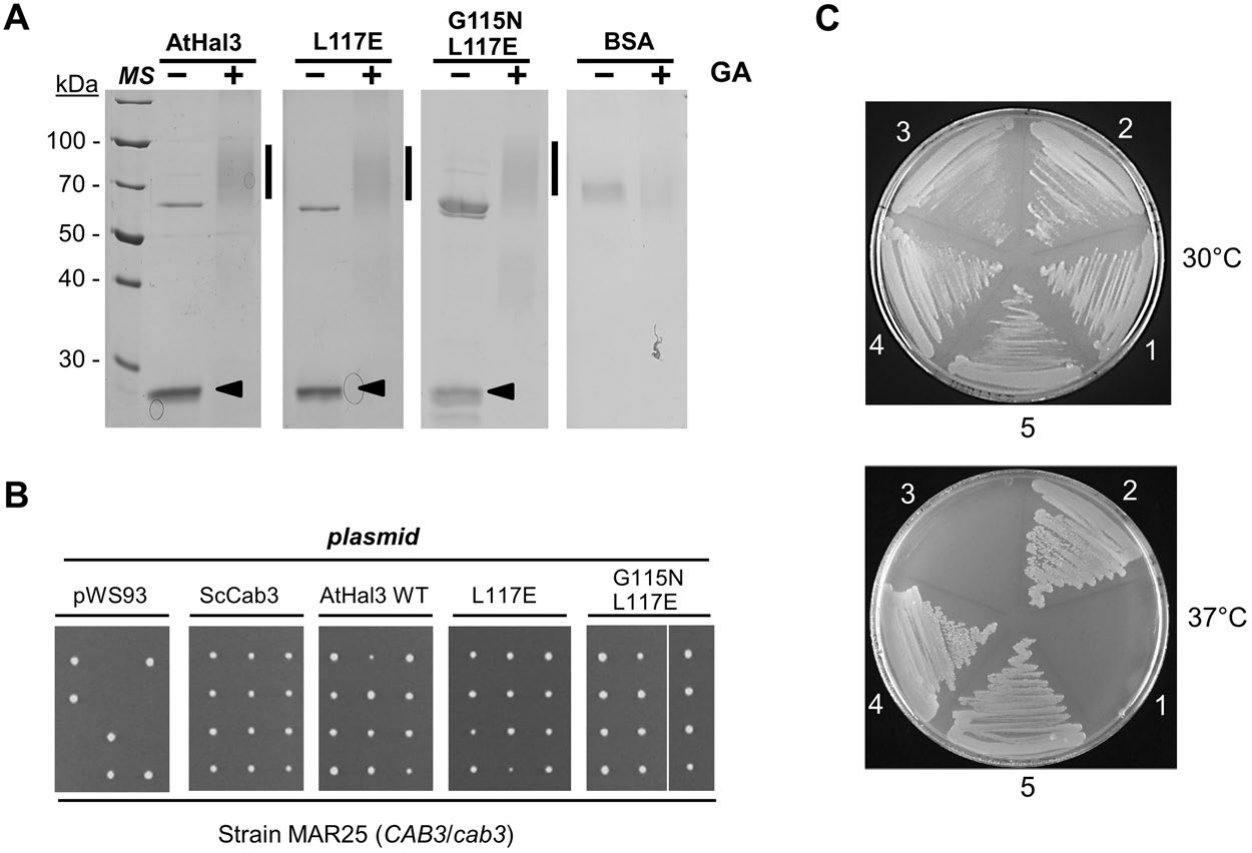
*In vitro* trimerization ability and *in vivo* PPCDC function of AtHal3 variants. (**A**) *In vitro* crosslinking of AtHal3 variants. Samples (1 μg) of native AtHal3a or its variants were incubated with 0.01% glutaraldehyde (GA, +) or treated with vehicle alone (−). Samples were resolved by SDS-PAGE (10% polyacrylamide gel) and proteins were visualized by staining with Coomassie Brilliant Blue R-250. The arrowhead denotes mobility of the untreated monomer and the vertical bar on the right indicates the region at ∼70–80 kDa (the expected size of a homotrimer). Note the presence of a ∼70 kDa contaminating protein of bacterial origin (DnaK). Original images can be found in Supplementary Fig. S3. (**B**) The indicated proteins were expressed from the pWS93 vector in strain MAR25, a diploid heterozygous for the *cab3* deletion (which is lethal in homozygosity). Upon sporulation, tetrads were dissected. Growth of all four spores indicates that the specific protein can replace *in vivo* Cab3 and, thus, likely conserve PPCDC function. (**C**) *E*. *coli* strain BW369 (*dfp-707*ts) was transformed with the empty plasmid pGEX6P-1 (1), or the same plasmid carrying native AtHal3 (as positive reference, 2), native ScHal3 (negative control, 3), AtHal3^L117E^ (4), and AtHal3^G115N L117E^ (5) and streaked on ampicillin-containing plates. Plates were incubated at 30 °C (permissive) or 37 °C (restrictive temperature) for 24 h.

We next investigated whether the mutations introduced in AtHal3 could affect the ability of this protein to form trimers *in vitro*. To this end, we expressed the recombinant proteins in *E*. *coli* as GST-fusions. Upon affinity purification, the GST-tag was removed and samples of each protein were treated with glutaraldehyde to covalently cross-link the possible trimeric complexes. As shown in Fig. 3A, treatment with glutaraldehyde resulted in all cases in the appearance of a broad band around 70–80 kDa, compatible with the generation of a AtHal3 trimer (theoretical molecular mass ∼69 kDa), indicating that none of the mutations affected the activity of AtHal3 to trimerize. Because the ability to form trimers is a requirement to generate an active PPCDC enzyme, we speculated that both mutated AtHal3 versions would be functional PPCDCs. To test this, we transformed strain MAR25, a diploid heterozygous for the *cab3* deletion, with all three constructs used in Fig. 2, induced sporulation of the cells and carried out tetrad analysis of the transformants (Fig. 3B). From previous studies it is known that native AtHal3 can functionally replace ScCab3, and therefore it led to the generation of four viable spores. The same behavior was found for both the single L117E and the double G115N L117E mutated variants. Similarly, transformation of a diploid heterozygous for the *hal3* and *vhs3* deletions (strain AGS4) allowed the recovery from parental ditype and tetratype tetrads of viable spores with the makers associated to the double chromosomal deletion.

These results indicated that none of the mutations interfered with the function of AHal3 in CoA biosynthesis. To test this further, we transformed the *E*. *coli* strain BW369 with plasmids bearing native Athal3 and its variants. This bacterial strain carries the *dfp-*707^ts^ mutation in the *dfp* gene (later renamed as *coaBC*) that abolishes PPCDC activity when cells are grown at 37 °C. As shown in Fig. 3C, the presence of the empty plasmid or the plasmid carrying ScHal3 did not allow growth of bacterial cells at 37 °C. In contrast, as previously described^12,14^ native AtHal3 complemented the *dfp* mutation. Interestingly, vigorous growth at the restrictive temperature was also observed for the AtHal3 L117E and G115N L117E variants, further confirming that these mutations do not alter normal PPCDC function.

### Functional characterization of ScHal3 variants as Ppz1 inhibitors *in vivo*

**Figure 4:**
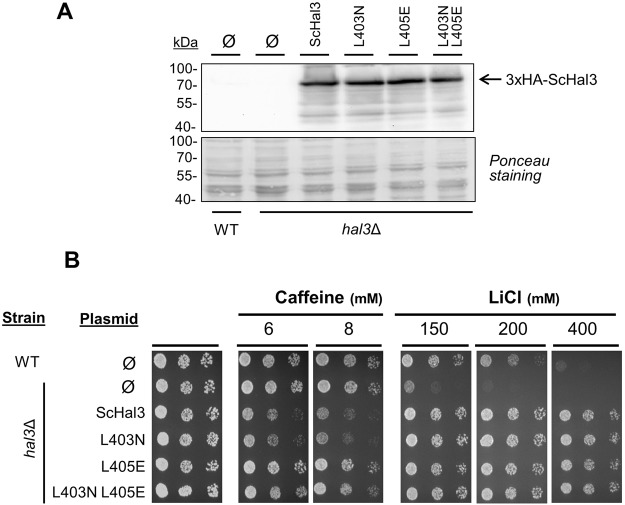
Immunodetection and phenotypic characterization of ScHal3 variants. (**A**) Wild type strain BY4741 (WT) and its *hal3Δ* derivative were transformed with plasmid pWS93 (Ø) and the same plasmid expressing native ScHal3 or the indicated variants. Protein extracts were prepared, subjected to SDS-PAGE and HA-tagged proteins detected as in Fig. 2. (**B**) Wild type (WT) or *hal3*Δ strains containing the indicated plasmids were spotted as in Fig. 2B on plates containing the indicated amounts of caffeine or LiCl. Plates were grown for 72 h.

We subsequently introduced similar mutations to ScHal3. Leu405 was mutated to Glu as was done for AtHal3, while the second Leu residue at position 403 (which is G115 in AtHal3) was mutated to Asn. This was done as Cab3, which has been shown not to be able to from homotrimers, has an Asn residue in the equivalent position. The variant containing both mutations was also constructed and all of them were expressed from plasmid pWS93 in *S*. *cerevisiae*. As can be observed in Fig. 4A, immunoblot analysis indicates that all variants are expressed roughly at the same level as native ScHal3. Phenotypic analysis of Hal3-deficient cells expressing the different forms of the protein showed (Fig. 4B) that cells expressing the L403N version did not differ from those expressing native ScHal3, that is, they showed decreased growth in presence of caffeine and increased tolerance to toxic Li^+^ ions. Interestingly, tolerance to caffeine of the cells carrying the single L405E or the double mutated ScHal3 was similar to that of cells carrying the empty plasmid. In contrast, all mutated versions increased tolerance to LiCl similarly to native ScHal3. These results indicated that the L405E mutation specifically affects certain functions of the protein and that, because the effect of ScHal3 on caffeine and Li^+^ tolerance is related to its inhibition of Ppz1, this is due to some impact on the ability to regulate the phosphatase.

### *In vitro* characterization of ScHal3 variants

**Figure 5:**
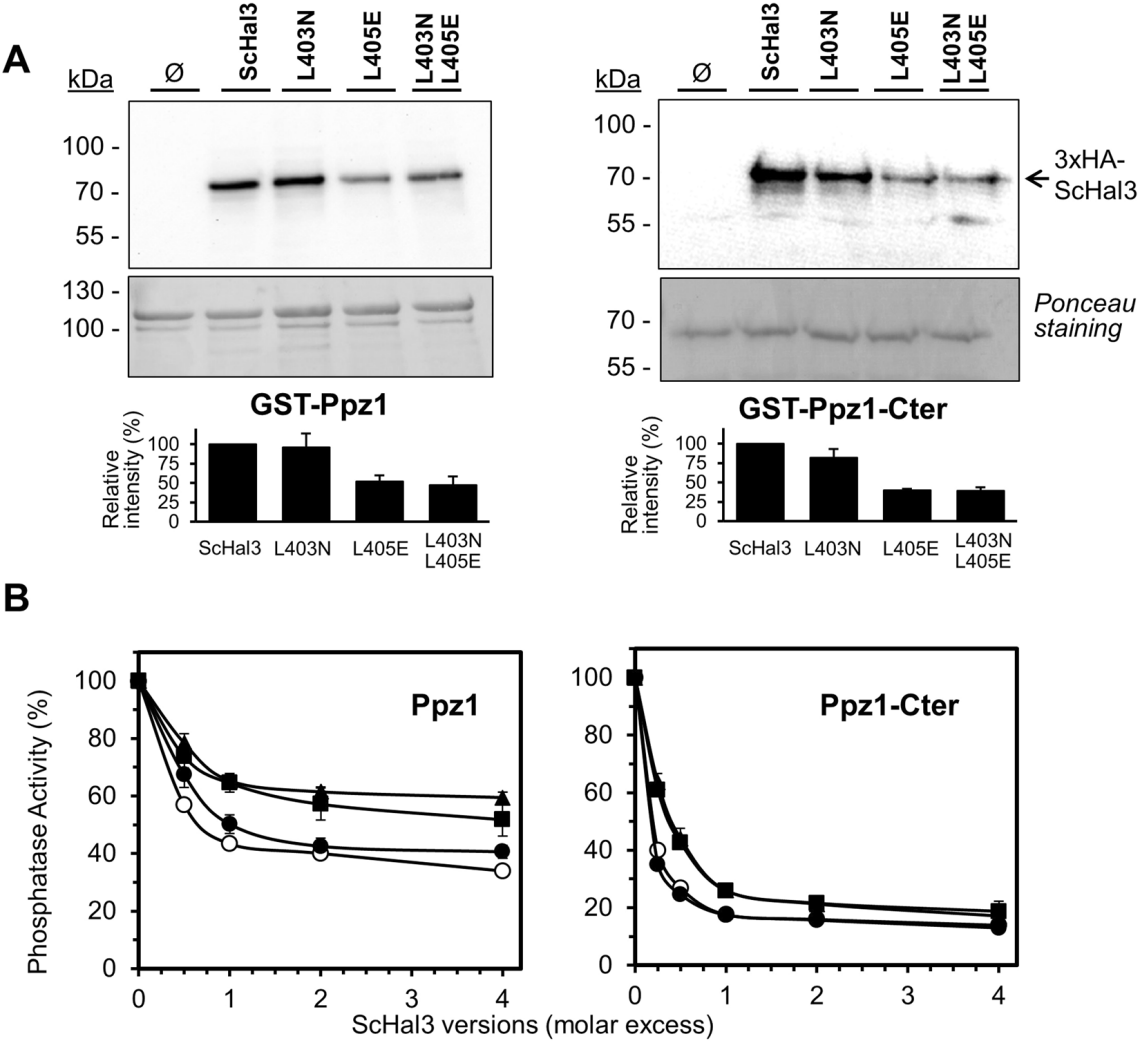
Ppz1 interaction and inhibitory capacity of ScHal3 variants. (**A**) Equal amounts of the indicated versions of the GST-tagged phosphatases were immobilized on glutathione beads and used as an affinity system to recover HA-tagged ScHal3 versions expressed from the pWS93 plasmid in IM021 (*ppz1 hal3*) cells. Beads were washed and processed for SDS-PAGE (10% gels) and immunoblotting was carried out using anti-HA antibodies as described in Methods. Ponceau staining of the membranes reveals the relative amounts of GST-Ppz1 or GST-Ppz1-Cter. The histograms represent the relative interaction ability of the mutated versions, quantified from 3 independent experiments (mean ± SD). (**B**) Recombinant Ppz1 or its C-terminal catalytic moiety (10 pmols) were incubated with increasing amounts of recombinant native ScHal3 (○) or its variants (●, L403N; ▲, L403N; ■, L403N L405E). Data are represented as percentage of the activity of the enzyme preparations in the absence of inhibitors and correspond to the mean ± SEM from 4 to 8 independent determinations using at least two different preparations of each protein.

We next tested how these mutations might affect the ability of ScHal3 to interact with and inhibit Ppz1. To this end, we set up *in vitro* interaction experiments using as bait recombinant GST-fused full length Ppz1 or its C-terminal catalytic domain (to which ScHal3 shows even stronger binding) bound to glutathione resin, and the diverse HA-tagged ScHal3 versions expressed in *S*. *cerevisiae* as preys. As shown in Fig. 5A, mutation of L403 to Asn barely affected interaction with GST-Ppz1. In contrast, the L405E and the double mutated variant showed decreased interaction, estimated to be about 50% compared to the native protein (Fig. 5A, lower panel). This defect was amplified when the C-terminal moiety of Ppz1 was used. We then expressed the ScHal3 variants in *E*. *coli* as GST-fusion proteins and upon removal of the GST-tag by protease treatment (Supplementary Fig. S1), used these proteins to test their ability to inhibit Ppz1 in an *in vitro* phosphatase assay. The results of this experiment showed (Fig. 5B), that the L403N mutation had no effect on the ability of ScHal3 to inhibit Ppz1 activity, whereas the proteins bearing the L405E mutation were clearly less effective as phosphatase inhibitors. The effect was more evident when the full-length phosphatase was used.

### Mutated Hal3 variants still can reconstitute PPCDC function in *S*. *cerevisiae*

**Figure 6:**
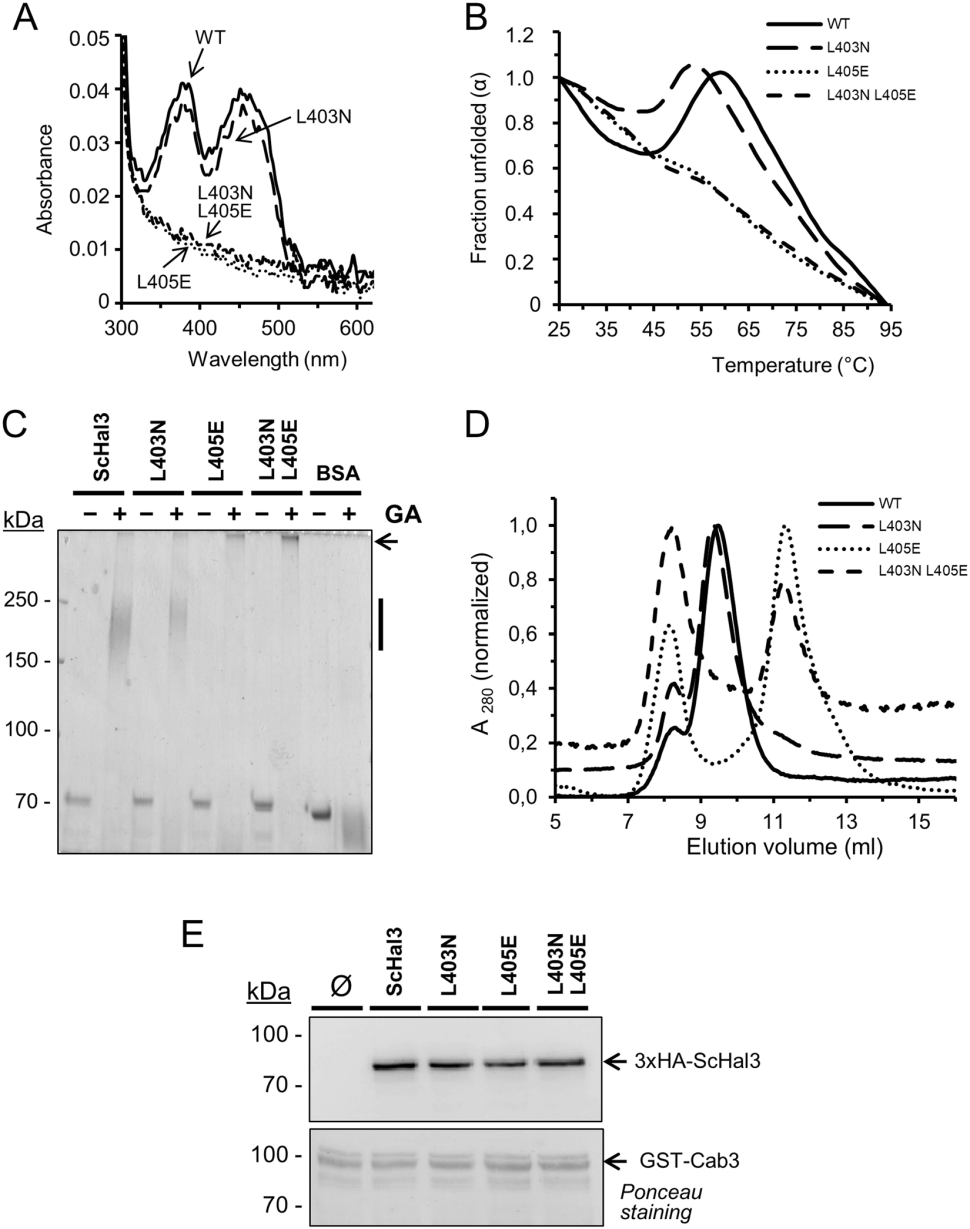
FMN content and trimerization ability of the different ScHal3 variants. (**A**) UV-Visible spectra of the indicated preparations showing the presence or absence of peaks at 382 and 452 nm. WT denotes native ScHal3. (**B**) Temperature-dependent structural stability analysis of ScHal3 variants. The temperature-dependent change in the normalized Trp-derived fluorescence values of native Hal3 and its mutated variants is shown. Wavelengths for excitation and emission were 295 nm and 338 nm, respectively, and the temperature was increased in steps of 2 °C. (**C**) *In vitro* cross-linking of ScHal3 variants. Samples (2 μg) were processed as in Fig. 3, resolved by SDS-PAGE (6% polyacrylamide gel) and proteins were visualized by staining with Coomassie Brilliant Blue R-250. The vertical bar on the right denotes the region in which proteins of a MW of ∼190 kDa (the predicted size of the homotrimer) is expected, while the arrow indicates the appearance of very high molecular mass structures. (**D**) The indicated samples were subjected to size exclusion chromatography and the A_280_ was monitored and normalized in each case to the highest value (taken as the unit) to account for the difference in concentration. (**E**) Ten µg of GST-Cab3 was immobilized on glutathione-sepharose beads and used as an affinity system to recover HA-tagged ScHal3 versions expressed in IM021 yeast cells. Beads were washed and processed for SDS-PAGE (8% gels) and immunoblotting was carried out using anti-HA antibodies as described in Methods. Ponceau staining of the membranes reveals similar amounts of GST-Cab3 in all lanes. Ø, empty plasmid.

It has been shown that the ability of ScHal3 to regulate Ppz1 can be dissociated from its role as component of the PPCDC heterotrimer. ScHal3 (as is the case with AtHal3) is a flavoprotein, and the presence of a FMN moiety, which is essential for the mechanism of PPCDC catalysis, confers a yellowish color to concentrated preparations of the protein. Interestingly, recombinant preparations of native ScHal3 and the L403N variant were yellow, but both versions carrying the L405E mutation were not, suggesting that they were unable to associate with FMN. This was confirmed by UV-visible scanning of preparations of all these proteins (Fig. 6A), which showed in the latter versions the absence of the characteristic peaks at 382 and 452 nm, indicative of the presence of oxidized flavin in the molecule. We considered that the failure to retain FMN could be due to structural changes induced by the L405E mutation and affecting trimerization ability. Indeed, the temperature-dependent fluorescence profiles of native Hal3 and the L403N variant were rather similar, with a decrease in fluorescence from 25 to 45 °C followed by a recovery at 55–60 °C and fast decline above 65 °C (Fig. 6B). In contrast, in both variants carrying the L405E mutation the emission of fluorescence continuously declined with the temperature. We finally tested the ability of the diverse ScHal3 versions to form trimers *in vitro*. The recombinant ScHal3 versions were cross-linked with glutaraldehyde, similarly to what was done for AtHal3 variants. The results (Fig. 6C) indicate that whereas the L403N mutation did not alter the ability of ScHal3 to form trimers, both versions containing the L405E change did not form typical trimers, but instead cross-linked giving rise to very high molecular mass complexes that barely entered into the polyacrylamide resolving gel. When a different cross-linker was used (BS2G), most of the protein corresponding to the L405E mutation remained in monomeric form (Supplementary Fig. S2). Finally, we subjected preparations of recombinant native ScHal3 and its variants to size exclusion chromatography. As shown in Fig. 6D, native ScHal3 and the L403 variant eluted together at a smaller volume than both variants carrying the L405E mutation, suggesting for the latter the presence of monomers (although their elution volume corresponded to species with somewhat larger size, possibly due to the non-globular shape of the N-terminal and C-terminal domains). It is worth noting that the versions carrying the L405E mutation also presented a prominent additional peak close to the V_0_, compatible with species of very high molecular mass. It is likely that these species correspond to those unable to enter into the resolving gel identified in the glutaraldehyde crosslinking experiments.

The results described above indicate that the L405E mutation compromises the ability of ScHal3 to retain the FMN moiety and to form standard trimeric complexes. These features would also be important for its function as a constituent of the heterotrimeric PPCDC complex. Therefore, we sought to test if the mutated variants could replace endogenous ScHal3 as components of an active PPCDC. To this end, we transformed the yeast strain ASG4, heterozygous for the *hal3* and *vhs3* deletions, with pWS93-based vectors expressing the diverse forms of ScHal3. Transformants were induced to sporulate and 18 to 35 tetrads were analyzed for the presence or absence of germinating spores giving rise to plasmid-bearing haploids cells carrying both *hal3 vhs3* mutations. To our surprise, we could recover this kind of events from non-parental ditype and tetratype tetrads, suggesting that, *in vivo*, ScHal3 variants carrying the L405E mutation can still associate with Cab3 and form functional PPCDC complexes. To confirm this conclusion further, we expressed recombinant Cab3 in *E*. *coli* as a GST-fusion protein and recovered the protein by affinity purification with glutathione-sepharose beads. Immobilized recombinant Cab3 was then used as a bait to test if the mutated ScHal3 forms, expressed in yeast, retained the ability to interact with Cab3. As shown in Fig. 6E, all mutated ScHal3 versions were retained by Cab3 with roughly equal affinity. This result supports the notion that the mutations do not impair the ability of ScHal3 to form functional heteromers with native Cab3 in yeast.

## Discussion

The ability of Hal3 to act as a moonlighting protein, participating as a constituent of the heterotrimeric PPCDC complex while also acting—in its monomeric form—as inhibitor of phosphatase Ppz1, suggest that Hal3 must be able to actively switch between its oligomeric states. Inspection of the trimeric structure of AtHal3 deduced from X-ray diffraction data and the model of the ScHal3 structure built upon it, suggested the possibility of a hydrophobic core involving G115 and L117 in plant Hal3 (L403 and L405 in the yeast Hal3) that is relevant for trimer stability.

In this study, we observe that mutations in AtHal3 G115 and/or L117 do not alter trimerization ability nor PPCDC function. Similarly, mutation of L403 in ScHal3 does not alter trimerization, suggesting is not pivotal for inter-monomer interaction. However, change of L405 (the residue equivalent to AtHal3 L117) avoids normal trimerization *in vitro* and produces very high MW species upon glutaraldehyde cross-linking. This could be explained by random cross-links affecting the large (∼290 residues) N-terminal extension of ScHal3 (that is not present in AtHal3), which is predicted to be unstructured. Such artefactual interactions could be enhanced by the failure to correctly interact within the PD region of ScHal3. Taken together, this data suggests that AtHal3 forms more stable trimers than ScHal3—most likely due to optimized interactions at the trimer interfaces—so that disruption of any interactions following mutation of G115 and/or L117 are of little relevance. Such a notion is supported by previous studies^18^ that reported the human PPCDC (HsCoaC), which is similar in size and sequence to AtHal3, to be much more stable than the PD (core domain) of ScHal3 based on having a melting temperature 20 to 30 °C higher.

In line with the results of the oligomer analysis, mutation of L403 also did not alter the behavior of ScHal3, suggesting that this residue has little functional relevance. In contrast, mutation of L405 to Glu has an important impact on ScHal3 features. First, the recombinant protein no longer retains FMN, suggesting that the ability to form standard trimers is lost (Fig. 6A). This conclusion is supported by the difference in the denaturation profiles of the native and mutated Hal3 variants, as those containing the L405E shows a gradual but steady loss of tertiary structure (as monitored by the loss of Trp fluorescence), while the native protein and the L403N mutant shows a curious biphasic profile (Fig. 6B). This profile most likely relates to denaturation taking place in two stages, the first being loss of the oligomeric structure (which we previously demonstrated is accompanied by loss of the FMN cofactor^18^), while the second relates to loss of tertiary structure. In these analyses, the profiles are likely the result of the total emission observed after interaction of the Trp and FMN fluorescence.

It is worth noting that in the case of AtHal3, the mutation L117E does not impair the protein’s ability to retain FMN (not shown), indicating again that this residue is dispensable for trimer formation. It has been reported that Cab3 itself can dimerize but do not form trimers^27^, and our own data indeed confirmed that recombinant Cab3 does not bind FMN (not shown). However, our results demonstrate that, while the ScHal3 L405E version cannot form standard homotrimers able to bind FMN, it is still able to substitute for native ScHal3 *in vivo* and contribute to the generation of functional PPCDC enzymes. Therefore, it must be assumed that the L405E variant is able to efficiently interact with Cab3 and reconstitute a functional FMN-containing catalytic site. Therefore, the inclusion of FMN in the complex is most probably the result of the interaction between both proteins. In fact, we demonstrate that recombinant Cab3 interacts *in vitro* with all ScHal3 variants as efficiently as with the native protein. It has been shown that *in vivo*, Cab3 interacts with other enzymes of the CoA biosynthetic pathway, such as Cab2, Cab4 and Cab5^27^. Such interactions might contribute to reconstitute a functional PPCDC catalytic site involving the L405E ScHal3 variant.

In a simplistic model, one would expect that the expression in yeast of ScHal3 versions unable to form standard trimers would increase the amount of free monomers, and that this would result in stronger inhibition of Ppz1. This, in turn, should lead to phenotypes reminiscent of those observed upon deletion of the phosphatase gene. However, we simply find a Hal3 loss of function when cells are challenged with caffeine, whereas overexpresion of the ScHal3 L405E variants increase tolerance to Li^+^ cations with potency similar to that of the native version (Fig. 4). Such behavior cannot be explained by different levels of expression, but by the partial inability of L405E variants to bind to Ppz1 and, consequently, by a noticeable decrease in inhibitory capacity (Fig. 5). Therefore, whereas change of L403 has no effect, replacement of L405 by Glu affects the ability of Hal3 to interact with Ppz1. Such a conclusion has important implications, as this interaction would only be relevant for monomeric Hal3, when this normally buried Leu becomes exposed. Such an observation provides further support to the notion that Hal3 interacts with Ppz1 in the monomeric state^18^.

It might appear surprising that, when tested for Ppz1-mediated functions, mutation of L405E yields a Hal3 protein that behaves as the native protein in some cases, but appears ineffective in others. However, such disparate behavior has been previously observed in specific Hal3 point-mutants^20^, when expressing heterologous Hal3-like proteins, as in the case of the *Schizosaccharomyces pombe* SpHal3^17^, or even when certain versions of Ppz1 carrying partial deletions of the N-terminal half were tested^28^. One possible explanation would be that the inability to inhibit Ppz1 fully affects certain Ppz1-related functions and not others. It is important to note that the role of Ppz1 in blocking the induction of *ENA1* was shown to be independent of its negative effect on K^+^ influx through Trks transporters^8^. The detrimental effect of Hal3 overexpression on caffeine (also observed in a *slt2* mutant) is likely due to the a strong inhibition of Ppz1, leading to excessive K^+^ influx and increase in cellular turgor^10^. Therefore, one could assume that full inhibition of Ppz1 would be needed to allow uncontrolled K^+^ entry, while partial inhibition would be sufficient to trigger *ENA1* expression and thus increase Li^+^ tolerance.

## Methods

### Yeast strains and growth conditions

Unless otherwise stated, *Saccharomyces cerevisiae* cells were grown, at 28 °C in YPD medium (10 g/L yeast extract, 20 g/L peptone, and 20 g/L dextrose) or in complete minimal medium (CM) lacking the appropriate requirements when carrying plasmids for selection^29^. All yeast strains used in this work are listed in Table 1.

**Table 1:** Yeast strains used in this work.

Strain	Genotype	Source/Reference
BY4741	*MAT* **a** *his3Δ1 leu2Δ0 met15Δ0 ura3Δ0*	^37^
*hal3 Δ*	BY4741 *hal3*::*KANMx*	^37^
DBY746	*MAT α his3Δ1 leu2Δ3 112 ura3-52 trp1-289 met15Δ0 ura3Δ0*	D. Botstein
EDN4	*DBY746 hal3*::*LEU2*	^38^
IM021	*MAT* **a** *ura3-52 leu2-3*, *112 trp1-1 his4 can-1r ppz1*::*KANMX hal3*::*LEU2*	^20^
MAR25	*MAT* **a**/*α ura3-52 leu2-3*, *112 trp1-1 his 4 can-1r CAB3*/*cab3*::*KANMX*	^12^
AGS4	*MAT* **a**/*α ura3-52 leu2-3*, *112 trp1-1 his 4 can-1r HAL3*/*hal3*::*LEU2 VHS3*/*vhs3*::*KANMX*	^6^

### DNA techniques and plasmid construction

*Escherichia coli* DH5α strain was used as plasmid DNA host and was grown in LB medium at 37 °C supplemented with 50 μg/ml ampicillin when needed for plasmid selection. *E*. *coli* strain BL21 (DE3) RIL was used for heterologous protein expression as described below. *E*. *coli* strain BW369, which carries a single-point temperature-sensitive mutation in the *coaBC* gene (*dfp*-707^ts^) that abolishes PPCDC activity at 37 °C has been previously described^30–32^. Restriction reactions, DNA ligations, and other standard recombinant DNA techniques, including bacterial and yeast transformations were performed using standard methods. Plasmid pGEX-AtHal3 expressing the wild type AtHal3 ORF was constructed by amplification of the AtHal3a cDNA from plasmid pRS699-AtHal3a^26^ with oligonucleotides AtHal3_EcoRI_5 and AtHal3_SalI_3. The amplification product was digested with EcoRI and SalI and cloned in these same sites of plasmid pGEX-6P-1 (GE Healthcare) and pWS93 (*ADH1* promoter, 2-micron, *URA3* marker)^33^. Mutated versions of AtHal3 and ScHal3 were made as follows. Plasmid pGEX-AtHal3^L117E^ was created using the pGEX-AtHal3 plasmid as template by the QuickChange Site-Directed Mutagenesis Kit (Stratagene), with Q5 High-Fidelity DNA Polymerase (New England Biolabs) and oligonucleotides AtHal3_L117E_5, and AtHal3_L117E_3. Plasmid pGEX-AtHal3^G115N L117E^ was made similarly from pGEX-AtHal3^L117E^ using oligonucleotides AtHal3_G115N_L117E_5 and AtHal3_G115N_L117E_3.

pGEX-ScHal3^L405E^ was also made by the QuickChange method from pGEX-ScHal3^20^ and oligonucleotides ScHal3_L405E_5 and ScHal3_L405E_3. In contrast, pGEX-ScHal3^L403N^ was made by 2-step PCR. In a first reaction, using pGEX-ScHal3 as template, two overlapping fragments were generated using the pairs of oligonuclotides 5HAL3_EcoRI/ScHal3 L403N 3′ and ScHal3 L403N 5′/3HAL3_XhoI. A mixture of both fragments was subsequently amplified using 5HAL3_EcoRI and 3HAL3_XhoI. The amplification fragment was cloned into the EcoRI and SalI sites of plasmid pGEX-6P-1. The same method was used to generate double mutations in ScHal3 using in this case pGEX-ScHal3^L405E^ as template. To introduce the L403N exchange, two PCR amplifications were carried out with primers 5′-HAL3 EcoRI/ScHal3_L403N_L405E_3 and ScHal3_L403N_L405E_5/3′-HAL3_XhoI. The second PCR was carried out with oligonuclotides 5′-HAL3_EcoRI and 3′-HAL3_XhoI, and the PCR fragment cloned into the EcoRI and XhoI sites of pGEX-6P-1. Construction of vectors for expression of GST-fused recombinant full length Ppz1 and the Ppz1-Cter (Δ1–344) catalytic moiety has been previously described^6^.

For expression in yeast the diverse inserts were released by digestion with EcoRI and XhoI and cloned into the EcoRI and SalI sites of plasmid pWS93. The construction of pWS93-Cab3 is described in^12^. All oligonucleotides used in this work are listed in Supplementary Table S1.

### Yeast protein extraction and immunoblot analysis

Yeast cells containing plasmids expressing the different versions of AtHal3 and ScHal3 were tested for integrity and expression levels of these proteins. For this purpose, *S*. *cerevisiae* BY4741 (wild type) and its isogenic derivative *hal3Δ* were transformed with the different constructs and cells were grown in 10 ml of synthetic minimal medium lacking uracil at 28 °C to A_660_ of ∼0.8. Protein extracts were prepared as described in^34^, and forty μg of total protein were resolved by SDS-PAGE. For immunobloting, proteins were transferred to Immobilon-P membranes (Millipore), and HA-tagged proteins were immunodetected using anti-HA primary antibody from Roche (1:1000 dilution) overnight, followed by the incubation with peroxidase conjugated mouse secondary antibody (GE Heathcare,1:20000 dilution) for one hour. Immunoreactive proteins were detected with ECL Prime (GE Healthcare) or Westar ηCUltra 2.0 (Cyanagen) kits.

### Recombinant expression of proteins in *E*. *coli*

All recombinant proteins were expressed in E. coli BL21 (DE3) RIL cells as described for Ppz1 in^35^ except that Terrific Broth was used instead of LB medium. GST-fused proteins were purified by glutathione-agarose affinity and, when required, the recombinant proteins were treated overnight at 4 °C with PreScission protease (GE Healthcare), following the manufacturer’s indications (80 units/ml resin), to cleave the GST moiety. The eluted GST-free proteins were analyzed by SDS-PAGE followed by Coomassie Blue-staining. The amount of each full-length recombinant protein was determined by scanning of the gel using Gel Analyzer software, integration of the appropriate bands and comparison with a calibration curve made with bovine serum albumin.

### Detection of protein-protein interaction

The ability of the diverse variants of ScHal3 to bind to Ppz1 was assessed *in vitro* by incubating aliquots (∼50 μl) of the glutathione-agarose beads containing 4 μg of GST-Ppz1 or GST-Ppz1-Cter with protein extracts (600 μg) prepared from strain IM021 (*ppz1 hal3*) transformed with plasmid pWS93 (negative control) and the same plasmid carrying the diverse HA-tagged version of ScHal3. Binding conditions and analysis were as in^16^. Forty μl of the final samples were subjected to SDS-PAGE and probed using anti-HA antibodies (Covance) as described above. Membranes were subsequently stained with Ponceau Red to monitor the amount of GST-Ppz1 loaded. Quantification of the interaction was carried out by calculating the ratio between each immunoreactive Hal3 signal and the corresponding stained phosphatase bait, and considering the value for native ScHal3 as the unit. At least two different phosphatase and regulator preparations were used.

The interaction of the different variants of ScHal3 with recombinant GST-Cab3 was evaluated in a similar way, except that glutathione-agarose beads containing 10 μg of GST-Cab3 and 2 mg of protein extracts derived from strain IM021 transformed with the ScHal3 variants were used. Forty μl of each sample were subjected to SDS-PAGE (8%), electroblotted and immunoreactive proteins revealed with anti-HA antibodies (Covance) as above.

Cross-linking of Hal3 variants with was carried out as in^17^ except that 0.01% glutaraldehyde was employed. For cross-linking using Bis-(sulfosuccinimidyl) glutarate (BS2G, Covachem), proteins (20 µg) were resuspended in the same buffer used for glutaraldehyde cross-linking at 0.2 µg/µl and incubated for one h at 25 °C with 200 µg of BS2G. Reaction was stopped by addition of ammonium bicarbonate at a final concentration of 100 mM and incubation for 10 min at 25 °C. Samples were analyzed by SDS-PAGE (6% gels) followed by Coomassie staining.

### Assay of protein phosphatase activity

The capacity of the different versions of Hal3 to inhibit full length Ppz1 or its catalytic domain was analyzed using recombinant proteins. To this end, the Ppz1 phosphatase activity was measured using *p*-nitrophenylphosphate as substrate essentially as described previously^16,36^ using 10 pmols of the phosphatase and 10 mM concentration of substrate. The phosphatase preparation was incubated with different amounts of the Hal3 variants for 5 min at 30 °C, and the assay was started immediately by the addition of the substrate.

### Fluorimetric analyses

Fluorimetric analyses were carried out with a FP-8200 Spectofluorometer (JASCO), using wavelengths of 295 nm and 338 nm for excitation and emission, respectively. The concentration of the GST-free recombinant proteins in the samples was 1 μM in a 10 mM Tris-HCl (pH 7.5), 150 mM NaCl, 10% glycerol and 2 mM DTT buffer. Thermal ramps were carried out at 2 °C/min.

### Other techniques

Tetrad analysis was carried out as in^19^. Asci were dissected using a MSM 300 Yeast Dissection Microscope (Singer Instruments). The genotype of haploid colonies derived from each spore was determined by replica on plates with selection media for the appropriate genetic markers. Fifteen to 29 tetrads were analyzed for AtHal3, and 18 to 35 tetrads for ScHal3 variants. The complementation test with the *dfp E*. *coli* mutant strain was done as in^12^. For size exclusion chromatography 500 μl (0.2–0.4 mg/ml) of the ScHal3 variants devoid of the GST moiety were loaded on a Superdex 200 HR 10/30 column and run in a Akta Explorer (GE Healthcare) chromatography equipment at a flow rate of 0.75 ml/min (1 ml fractions) with a buffer consisting of 10 mM Tris-HCl (pH 7.5), 150 mM NaCl, and 2 mM DTT.

## Electronic supplementary material

Supplementary Information

## Data Availability

All data generated or analyzed during this study are included in this published article (and its Supplementary Information files). Protocols, DNA constructs, and other materials generated in this work will be made available without restrictions upon request to the corresponding author.
