# Supplementary material for: Mutations at the hydrophobic core affect Hal3 trimer stability, reducing its Ppz1 inhibitory capacity but not its PPCDC moonlighting function
Source: Sci Rep. 2018 Oct 2;8:14701. doi: 10.1038/s41598-018-32979-x (PMC6168597; doi:10.1038/s41598-018-32979-x)
Supplement: Supplementary file 1 — Supplementary Information [file 41598_2018_32979_MOESM1_ESM.pdf]

Mutations at the hydrophobic core affect Hal3 trimer stability, reducing its Ppz1 inhibitory capacity but not its PPCDC moonlighting function.

Carlos Santolaria, Diego Velázquez, Erick Strauss and Joaquín Ariño

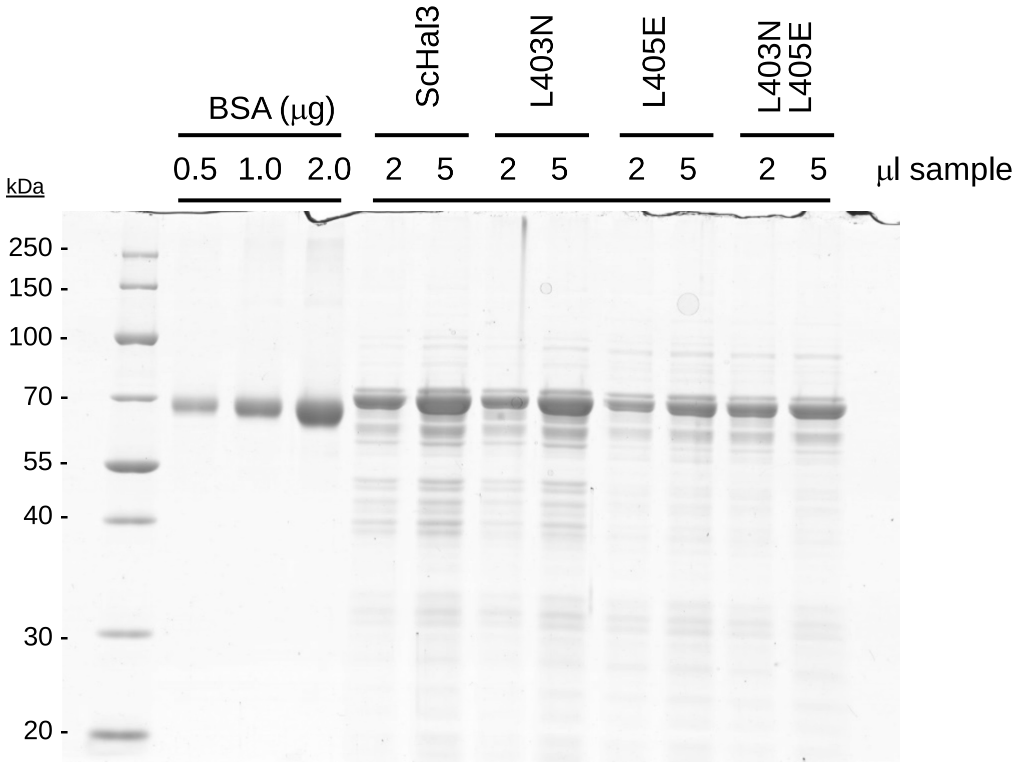

Supplementary Figure S1. Expression of ScHal3 and its variants in *E. coli* and, upon removal of the GST-moiety, analysis of the recombinant proteins (2 and 5 µl) by SDS-PAGE (10% polyacrylamide) followed by Coomassie staining. Bovine serum albumin (0.5 to 2.0 µg) was run in parallel for comparison of protein amounts.

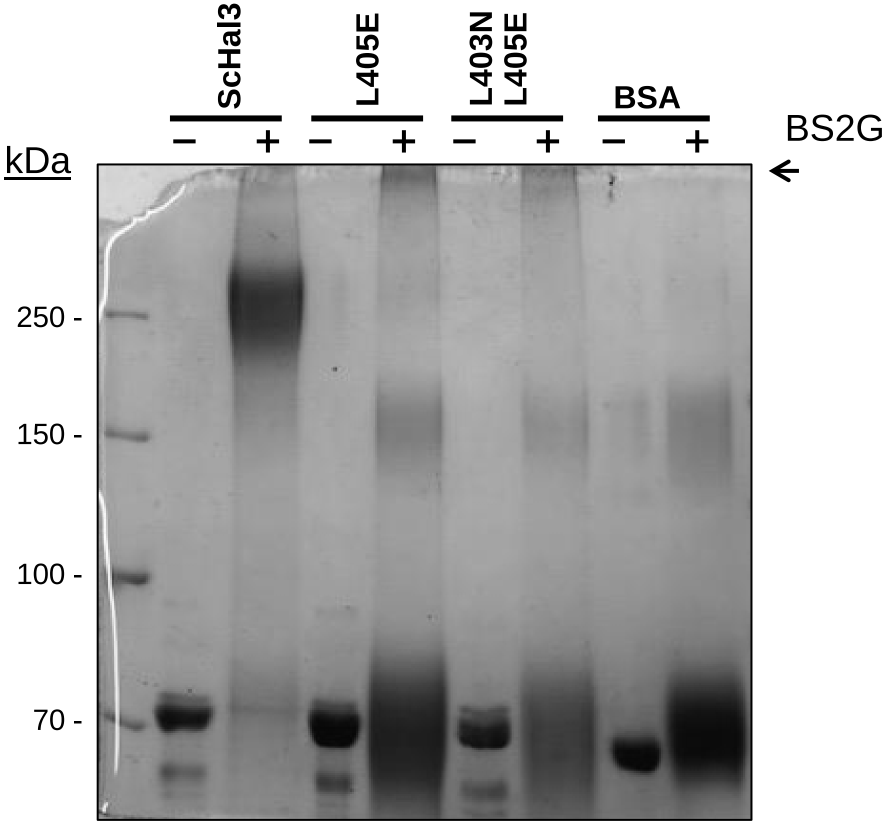

Supplementary Figure S2. *In vitro* cross-linking of ScHal3 variants using Bis-(sulfosuccinimidyl) glutarate (BS2G) Samples (20 µg) were processed as described in Material and Methods, resolved by SDS-PAGE (6% polyacrylamide gel) and proteins were visualized by staining with Coomassie Brilliant Blue R-250. Note that native ScHal3 forms species migrating with an apparent molecular mass of around 3-fold that of the monomers. The arrow indicates the appearance of very high molecular mass structures. The diffuse band appearing around 150 kDa is unspecific, as it also appears with BSA is treated with the cross-linking reagent.

Supplementary Table S1. Oligonucleotides used in this work

Name	Sequence	Description / Use
AtHal3_EcoRI_5	CGTGAATTCGAGAATGGGAAAAGAGACAG	AtHal3 cloning
AtHal3_SalI_3	TACGTCGACTTAACTAGTTCCACCGGTTT	AtHal3 cloning
AtHal3_L117E_5	AAGATTGCTGGTGGGGAATGTGATAATCTTCTGACTTGC	AtHal3 L117E mutation
AtHal3_L117E_3	TCAGAAGATTATCACATTCCCCACCAGCAATCTT	AtHal3 L117E mutation
AtHal3_G115N_L117E_5	CCTTAGGCAAGATTGCTAATGGGGAATGTGATAATC	AtHal3 G115N and L117E mutation
AtHal3_G115N_L117E_3	GATTATCACATTCCCCATTAGCAATCTTGCCTAAGG	AtHal3 G115N and L117E mutation
ScHal3_L405E_5	AAAATTGCTTTGGGCGAATGCGACAATCTTCTGACAA	ScHal3 L405E
ScHal3_L405E_3	CAGAAGATTGTCGCATTCGCCCAAAGCAATTTTTGATAA	ScHal3 L405E
ScHal3 L403N 5'	TTATCAAAAATTGCTAATGGCCTGTGCGACAATCT	ScHal3 L403N mutation
ScHal3 L403N 3'	GATTGTCGCACAGGCCATTAGCAATTTTTGATAATG	ScHal3 L403N mutation
ScHal3_L403N_L405E_5	TTATCAAAAATTGCTAATGGCGAATGCGACAATCT	ScHal3 L403N and L405E mutation
ScHal3_L403N_L405E_3	GATTGTCGCATTCGCCATTAGCAATTTTTGATAATG	ScHal3 L403N and L405E mutation
5HAL3_EcoRI	GGGAATTCATGACTGCCGTCGCCTCTACT	PCR for ScHal3 cloning in pGEX-6P1
3HAL3_XhoI	CACGCTCGAGCATAGACTATTTATTGATGC	PCR for ScHal3 cloning in pGEX-6P1

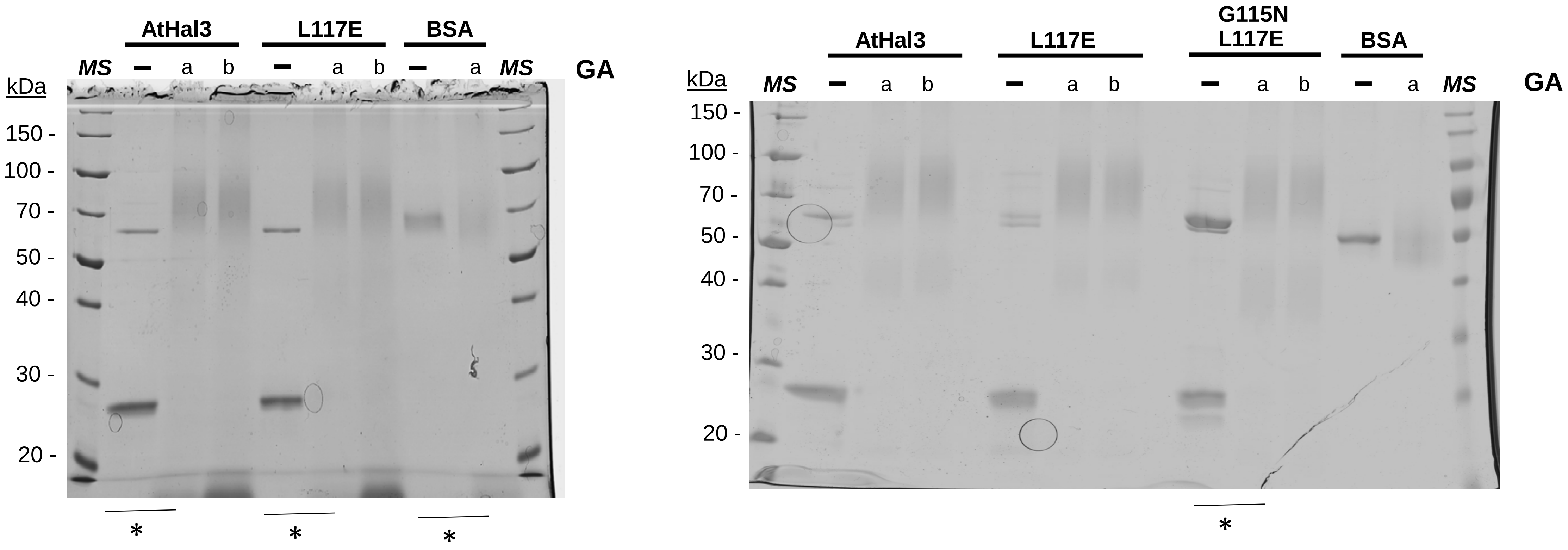

Supplementary Figure S3. Original uncropped images corresponding to those shown in figure 3A (denoted by asterisks at the bottom of the images. Concentration of glutaraldehyde (GA) used were: none (-), 0.01% (a) and 0.005% (b).
